# Comparison of Detection Yields Between Texture and Color Enhancement Imaging Mode 1 and Mode 2 for Colorectal Lesions: A Post‐hoc Analysis of a Multicenter Observational Study

**DOI:** 10.1002/deo2.70248

**Published:** 2026-01-20

**Authors:** Kensuke Shinmura, Hiroaki Ikematsu, Taku Sakamoto, Maasa Sasabe, Tatsuro Murano, Yasuhiko Mizuguchi, Hiroyuki Takamaru, Toshiki Futakuchi, Naoto Tamai, Kazuki Sumiyama, Yutaka Saito

**Affiliations:** ^1^ Department of Gastroenterology and Endoscopy National Cancer Center Hospital East Chiba Japan; ^2^ Division of Science and Technology for Endoscopy National Cancer Center Hospital East Chiba Japan; ^3^ Endoscopy Division National Cancer Center Hospital Tokyo Japan; ^4^ Department of Endoscopy The Jikei University School of Medicine Tokyo Japan

**Keywords:** colorectal neoplasia, detection, texture and color enhancement imaging mode 1, texture and color enhancement imaging mode 2, white light imaging

## Abstract

**Objectives:**

Colonoscopy is a reliable technique for the detection, diagnosis, and treatment of adenomas and early cancer. Image‐enhanced endoscopy (IEE) is important for detecting colorectal lesions. Texture and color‐enhancement imaging (TXI) has recently emerged as a novel modality for IEE. Thus, TXI operates in two modes: mode 1 (TXI1) enhances the structure, color, and brightness, whereas mode 2 (TXI2) does not. We have previously reported the detection of colorectal adenomas using TXI. We aimed to determine the detection yields of TXI1 and TXI2 using the data from our previous study.

**Methods:**

We retrospectively analyzed the colonoscopy data from three institutions between August 2020 and January 2021. The patients were classified into two groups: TXI1 and TXI2. The mean number of adenomas detected per procedure (MAP), adenoma detection rate (ADR), and flat adenoma detection rate (FDR) were compared between groups.

**Results:**

The evaluations (95% confidence intervals) for the TXI1 versus TXI2 groups were as follows: MAP, 1.5 (1.3–1.7) versus 1.5 (1.3–1.7); ADR, 56.8% (47.3–65.9) versus 59.7% (50.3–68.6); and FDR, 68.6% (59.5–76.9) versus 63.9% (54.6–72.5), with no statistically significant differences between the groups.

**Conclusion:**

The detection rates of colorectal lesions were comparable between the TXI1 and TXI2 groups.

## 1 Introduction

Total colonoscopy is considered the gold standard for detecting colorectal neoplasms such as colorectal adenomas and cancers. Total colonoscopy with polyp resection reduces the incidence and mortality of colorectal cancer [1, 2]. However, there have recently been concerns regarding post‐colonoscopy colorectal cancer (PCCRC), which refers to cancers detected after colonoscopy, and suggests the involvement of missed lesions. Reports indicate that total colonoscopy using white light imaging (WLI) has a miss rate for neoplastic lesions of approximately 20%–30% [3, 4, 5]. Various techniques, such as repeated and inverted observations and the use of distal attachments, have been implemented to avoid missing lesions and improve the detection of colorectal lesions. In addition, several studies have shown that image‐enhanced endoscopy (IEE) improves lesion detection [6, 7, 8, 9, 10, 11, 12]. Texture and color‐enhancement imaging (TXI) has recently emerged as a novel modality for IEE.

**FIGURE 1:**
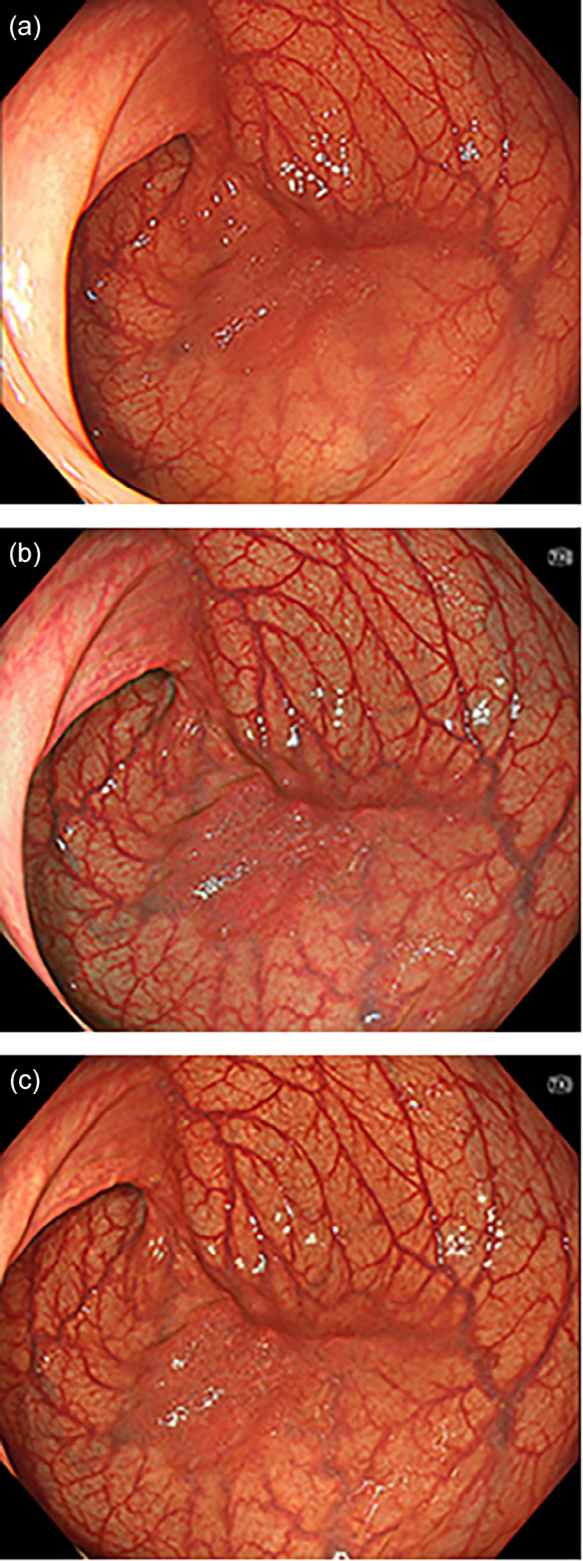
A 25‐mm LST‐NG in the ascending colon detected by each observation mode. (a) The lesion appears reddened under WLI, but its boundaries are not clearly defined. (b) In TXI1, the redness of the lesion is enhanced, and the boundaries of the lesion are clearly defined. (c) In TXI2, the contrast is enhanced compared to WLI, making the entire lesion appear clearer than with WLI. LST‐NG: Laterally spreading tumor‐non‐granular type, TXI: Texture and color‐enhancement imaging.

TXI has several key features, including enhanced red contrast, which helps distinguish slight color differences between lesions and normal mucosa [13, 14, 15]. TXI has two modes, TXI1 and TXI2, as shown in Figure 1. TXI1 is a color‐enhanced version of TXI2, and TXI1 has more contrast than WLI or TXI2 [16].

Some investigations on TXI have compared the visibility between TXI1 and TXI2 based on color differences, often reporting that TXI1 tends to provide higher visibility [14, 17]. However, the visibility scores of each IEE modality, including TXI, can vary depending on the endoscopist's experience [15]. Although our previous multicenter observational study demonstrated the overall benefit of TXI in polyp detection, the roles of TXI1 and TXI2 remain unclear [18]. A multicenter randomized controlled trial (RCT) clarified that the adenoma detection rate (ADR) was significantly higher in the TXI group than in the WLI group, and the number of adenomas per colonoscopy (APC) in the TXI group was significantly higher than that in the WLI group [16]. This RCT demonstrated the superiority of TXI over WLI; however, neither the original study nor the RCT mentioned the TXI modes used. As these two modes differ in their color‐enhancement algorithms, it is clinically meaningful to investigate whether this affects the detection yield in real‐world settings.

In this supplementary study, we aimed to clarify the detection yields of TXI1 and TXI2 using data from our original study [18].

## 2 Methods

### 2.1 Study Design

This retrospective study was designed as a post‐hoc analysis of a multicenter observational study on the detection of colorectal adenomas using TXI [18]. The original study was conducted at three Japanese institutions and was approved by the Institutional Review Board (IRB Approval No. 2020‐313). This study was conducted in accordance with the Japanese Ethical Guidelines for Medical and Health Research on Human Subjects. Informed consent was obtained from all participants at each participating institution using the opt‐out method.

### 2.2 Study Population

Data, including patient and lesion characteristics and colonoscopy findings, were collected from the patients’ medical records between August 2020 and January 2021. The study analyzed 237 patients (599 lesions) who met the following criteria: i) patients aged ≥ 20 years; ii) patients who underwent colonoscopy using the new endoscopy system; iii) indications for colonoscopy, including colorectal cancer screening (e.g., positive fecal occult blood test result), post‐treatment surveillance, a work‐up for lower gastrointestinal symptoms (e.g., melena, hematochezia, and other symptoms), polyp follow‐up, and pretreatment work‐up; iv) all lesions detected during colonoscopy were diagnosed histopathologically, excluding those that did not clinically require resection; v) repeated ascending colon observation, which was recommended by the Japanese Colonoscopy Screening and Surveillance Guidelines [19]; and vi) the second observation of the ascending colon was performed using TXI (TXI1 or TXI2) or WLI mode, which was not used in the first observation of the entire colorectum. This study was limited to patients for whom TXI was administered during the first observation. The exclusion criteria were as follows: i) patients with a history of colorectal resection; ii) inadequate bowel preparation, as evaluated using the Aronchick scale (Excellent, Good, Fair, Poor, Inadequate) [20, 21]; and iii) those who were assessed by the investigators as inappropriate for analysis.

### 2.3 Endoscopist and Colonoscopy

The endoscopists were classified as experts or non‐experts, with experts defined as those who taught endoscopic techniques to non‐experts at each institution. Each endoscopist determined the mode of observation during withdrawal. All procedures were performed using a CV‐1500 video processor system (EVIS X1; Olympus Medical Systems Corp., Tokyo, Japan), a newly developed colonoscope (CF‐EZ1500DI; Olympus Medical Systems Corp.), or a conventional endoscope (CF‐HQ290ECI, CF‐HQ290I, CF‐HQ290ZI, PCF‐H290TI, PCF‐H290ZI, or GIF‐H290T; Olympus Medical Systems Corp.).

### 2.4 Outcomes

The outcomes were as follows: mean number of adenomas detected per procedure (MAP), defined as the total number of adenomas divided by the total number of colonoscopies; ADR, defined as the proportion of patients with at least one adenoma or cancer; polyp detection rate (PDR), defined as the proportion of patients with at least one polyp; flat polyp detection rate (FDR), defined as the proportion of patients with at least one non‐polypoid polyp; and mean number of polyps detected per procedure (MPP), defined as the total number of polyps divided by the total number of colonoscopies. The lesions were detected during the first observation of the colorectum. Lesions detected during the second observation were excluded from the post‐hoc analysis. The ADR, PDR, FDR, MAP, and MPP were calculated for each patient. The outcomes were analyzed for each observation mode. All polyps were classified by location, size, and morphology according to the Paris classification [22, 23] and by histopathology. In this study, we defined 0–IIa, 0–IIb, 0–IIc, 0–IIa + 0–IIc, and 0–Is + 0–IIa as non‐polypoid polyps.

### 2.5 Histopathology Review

All polyps were reviewed by histopathologists at each institution. All lesions were diagnosed according to the Japanese Classification of Colon and Rectal Cancers [24]. Therefore, according to the World Health Organization classification, intramucosal carcinoma corresponding to high‐grade dysplasia and high‐grade mucosal neoplasia was defined as cancer.

### 2.6 Statistical Analysis

No procedures were performed to compensate for missing data. All statistical analyses were performed using SAS version 9.4 (SAS Institute, Cary, NC, USA) and Stata version 18 (StataCorp, College Station, TX, USA). Descriptive analyses were performed using means and standard deviations for continuous variables, and percentages for categorical variables. Differences in continuous variables were assessed using Student's t‐test, while categorical variables were compared using the chi‐square test. P‐values were calculated, and a two‐sided p < 0.05 was considered statistically significant. Point estimates and 95% confidence intervals (CIs) were calculated for the MAP, ADR, PDR, FDR, and MPP. The distributions used to construct the CIs for individual estimates were as follows: a Poisson distribution was used for MAP and MPP, and an F‐distribution (Clopper–Pearson exact CI) was used for ADR, PDR, and FDR. The CIs for the estimates were obtained using a two‐sided 95% CI. For MAP, *p*‐values were calculated using a Poisson regression model (Wald test) with multiplicity adjustment using the Tukey method. For ADR, PDR, and FDR, *p*‐values were calculated using a logistic regression model (Wald test) with multiplicity adjustment using Tukey's method.

In this post‐hoc analysis, we extended the original study's evaluation of TXI by further subdividing the TXI group into TXI1 and TXI2. We evaluated each outcome in the TXI1 and TXI2 groups, and the MAP was analyzed according to morphological type and lesion size. Furthermore, all outcomes were calculated according to the expertise levels of the endoscopists (expert and non‐expert).

## 3 Results

### 3.1 Patient and Procedural Characteristics

The patient characteristics are shown in Table 1. The TXI1 and TXI2 groups contained 118 and 119 patients, respectively. Post‐treatment surveillance colonoscopies were more frequent in the TXI1 group than in the TXI2 group, with 67 (56.8%) and 59 (49.6%) patients, respectively, although the difference was not statistically significant. Colonoscopies were performed by 11 experts and 24 non‐experts (Table 2). The patient and procedural characteristics did not differ significantly between the TXI1 and TXI2 groups.

**TABLE 1:** Patient characteristics.

Characteristics of patients	TXI1, *N* (%)	TXI2, *N* (%)	*p*‐Value
	118 (100)	119 (100)	
Age (mean, SD)	62.9 (12.4)	65.5 (11.8)	0.10
Sex, Male	76 (64.4)	78 (65.5)	0.92
Drinking history, Yes	32 (27.1)	41 (34.5)	0.22
Smoking history, Yes	48 (40.7)	55 (46.2)	0.39
Family history of CRC, Yes	21 (17.8)	19 (16.0)	0.71
Indications for colonoscopy			
Screening, including FIT positives	27 (22.9)	31 (26.1)	0.83
Post‐treatment surveillance	67 (56.8)	59 (49.6)	
Abdominal symptoms	1 (0.8)	2 (1.7)	
Polyp follow‐up	3 (2.5)	3 (2.5)	
Pretreatment work‐up	20 (16.9)	24 (20.2)	

CRC, colorectal cancer; FIT, fecal immunochemical test; polyp follow‐up, colonoscopy for patients with residual polyps that did not need to be removed; post‐treatment surveillance, colonoscopy for patients who had one or more neoplastic polyps that were completely removed during baseline colonoscopy; pretreatment work‐up, colonoscopy before treating colorectal polyps; SD, standard deviation; TXI, texture, and color enhancement imaging.

**TABLE 2:** Procedural characteristics.

Characteristics	TXI1	TXI2	*p*‐Value
Number of procedures, *N* (%)	118 (100)	119 (100)	
Aronchick scale, *N* (%)			
Excellent	64 (54.2)	69 (58.0)	0.51
Good	47 (39.8)	46 (38.7)	
Fair	5 (4.2)	4 (3.4)	
Poor	2 (1.7)	0 (0.0)	
Other	0 (0.0)	0 (0.0)	
Unknown	0 (0.0)	0 (0.0)	
Cecal intubation, *N* (%)			
Yes	118 (100)	119 (100)	1.0
Withdrawal time (min) Mean (SD),	8.7 (3.0)	8.6 (3.3)	0.81
Endoscopist, *N* (%)			
Expert	72 (61.0)	84 (70.6)	0.12
Non‐expert	46 (39.0)	35 (29.4)	

SD, standard deviation; TXI, texture and color enhancement imaging.

### 3.2 Lesion Characteristics

The lesion characteristics, including location, morphology, and size, did not differ significantly between the two groups (Table 3).

**TABLE 3:** Lesion characteristics.

		TXI1 *N* (%)	TXI2 *N* (%)	*p*‐Value
Location	Number of lesions	300 (100)	299 (100)	
	Ileum	0 (0.0)	0 (0.0)	0.11
	Cecum	22 (7.3)	18 (6.0)	
	Ascending colon	81 (27.0)	89 (29.8)	
	Transverse Colon	80 (26.7)	84 (28.1)	
	Descending colon	30 (10.0)	18 (6.0)	
	Sigmoid colon	65 (21.7)	53 (17.7)	
	Rectum	21 (7.0)	36 (12.0)	
	Anal canal	0 (0.0)	0 (0.0)	
Endoscopic diagnosis	Number of lesions	300 (100)	299 (100)	
	Adenoma, early cancer	221 (73.7)	221 (73.9)	0.75
	Advanced cancer	1 (0.3)	3 (1.0)	
	Serrated lesion	35 (11.7)	36 (12.0)	
	Others	43 (14.3)	39 (13.0)	
Morphologic type	Number of lesions	279 (100)	282 (100)	
	Polypoid	55 (19.7)	60 (21.3)	0.33
	Non‐polypoid	218 (78.1)	220 (78.0)	
	Others	6 (2.2)	2 (0.7)	
Size, mm	Number of lesions	272 (100)	281 (100)	
	≤5	186 (68.4)	185 (65.8)	0.79
	6–9	53 (19.5)	61 (21.7)	
	≥10	33 (12.1)	35 (12.5)	
Histopathology	Number of lesions	278 (100)	276 (100)	
	Adenoma	186 (66.9)	174 (63.0)	0.47
	TSA	1 (0.4)	0 (0.0)	
	SSL	15 (5.4)	9 (3.3)	
	Hyper	30 (10.8)	36 (13.0)	
	Cancer	8 (2.9)	10 (3.6)	
	Others	38 (13.7)	47 (17.0)	

SSL, sessile serrated lesion; TSA, traditional serrated adenoma; TXI, texture, and color enhancement imaging.

### 3.3 Detection Yield of Colorectal Lesion

The MAP, ADR, PDR, FDR, and MPP did not differ significantly between the TXI1 and TXI2 groups (MAP: 1.5 vs. 1.5; ADR: 56.8% vs. 59.7%; PDR: 80.5% vs. 79.8%; FDR: 68.6% vs. 63.9%; MPP: 2.3 vs. 2.3, respectively) (Table 4). The MAP for non‐polypoid lesions was 1.05 in the TXI1 group and 1.06 in the TXI2 group, with no significant difference (Table 5). The MAP for lesions measuring 6–9 mm and ≥ 10 mm was also comparable between the TXI1 and TXI2 groups (0.33 vs. 0.31 and 0.14 vs. 0.19, respectively).

**TABLE 4:** Comparison of the detected lesions between TXI1 and TXI2.

	TXI1		TXI2		*p*‐Value
	Point estimate	95% CI	Point estimate	95% CI	
MAP	1.5	1.3–1.7	1.5	1.3–1.7	1.0
ADR, %	56.8	47.3–65.9	59.7	50.3–68.6	0.89
PDR, %	80.5	72.2–87.2	79.8	71.5–86.6	0.99
FDR, %	68.6	59.5–76.9	63.9	54.6–72.5	0.72
MPP	2.3	2.1–2.6	2.3	2.1–2.6	1.0

ADR, adenoma detection rate; CI, confidence interval; FDR, flat polyp detection rate; MAP, mean number of adenomas detected per procedure; MPP, mean number of polyps detected per procedure; PDR, polyp detection rate; TXI, texture, and color enhancement imaging.

**TABLE 5:** Mean number of adenomas detected per procedure (MAP) by morphologic type and size of lesion.

		TXI1		TXI2		*p*‐Value
		Point estimate	95% CI	Point estimate	95% CI	
Morphologic type	Polypoid	0.356 (42/118)	0.263–0.482	0.395 (47/119)	0.297–0.526	0.62
	Non‐polypoid	1.051 (124/118)	0.881–1.253	1.059 (126/119)	0.889–1.261	0.95
	Others	0.025 (3/118)	0.008–0.079	0/119	−	N/A
Size, mm	≤5	0.915 (108/118)	0.758–1.105	0.933 (111/119)	0.774–1.123	0.89
	6–9	0.331 (39/118)	0.241–0.452	0.311 (37/119)	0.225–0.429	0.79
	≥10	0.136 (16/118)	0.083–0.221	0.185 (22/119)	0.122–0.281	0.35

CI, confidence interval; MAP, mean number of adenomas detected per procedure; TXI, texture and color enhancement imaging; N/A, not available.

As shown in Table 6, among experts and non‐experts, the outcomes (MAP, ADR, PDR, FDR, and MPP) did not differ significantly between the TXI1 and TXI2 groups.

**TABLE 6:** Comparison of outcomes by expert and non‐expert.

		TXI mode 1		TXI mode 2		*p*‐Value
		Point estimate	95% CI	Point estimate	95% CI	
Expert	MAP	1.6	1.3–2.0	1.4	1.2–1.8	0.63
	ADR, %	55.4	42.5–67.7	56.9	44.0–69.2	0.98
	PDR, %	76.9	64.8–86.5	76.9	64.8–86.5	1.0
	FDR, %	67.7	54.9–78.8	61.5	48.6–73.3	0.74
	MPP	2.5	2.1–2.9	2.2	2.0–2.6	0.67
Non‐expert	MAP	1.3	1.0–1.7	1.5	1.2–1.9	0.62
	ADR, %	58.5	44.1–71.9	63	48.7–75.7	0.88
	PDR, %	84.9	72.4–93.3	83.3	70.7–92.1	0.97
	FDR, %	69.8	55.7–81.7	66.7	52.5–78.9	0.94
	MPP	2.2	1.8–2.6	2.4	2.1–2.9	0.66

ADR, adenoma detection rate; CI, confidence interval; FDR, flat polyp detection rate; MAP, mean number of adenomas detected per procedure; MPP, mean number of polyps detected per procedure; PDR, polyp detection rate; TXI, texture, and color enhancement imaging.

## 4 Discussion

To our knowledge, this is the first report to compare colorectal lesion detection in TXI1 and TXI2 groups using data from a multicenter observational study [18]. In this post hoc analysis, the detection rates of colorectal lesions were comparable between the TXI1 and TXI2 groups. Additionally, we demonstrated that both experts and non‐experts achieved similar detection yields, regardless of whether TXI1 or TXI2 was used. These findings suggest that, in clinical practice, the choice between TXI1 and TXI2 may not substantially influence detection performance.

Many studies on lesion visibility or detection using TXI have been conducted in the upper and lower gastrointestinal fields [14, 15, 17, 25, 26, 27]. In both fields, TXI provided a higher lesion visibility than WLI. Therefore, TXI is expected to improve the detection of colorectal lesions. After our original study, some studies demonstrated an improvement in colorectal lesion detection with TXI and reported detection yields of TXI1 and TXI2 [16, 28, 29, 30, 31]. In the RCT by Young et al., for TXI1, the ADR and MAP were 54.6% and 1.71, respectively [30]. Regarding TXI2, an RCT by Pattarajierapan et al. reported an ADR of 52.1% and a MAP of 1.2 [29]. Although the present study was retrospective, with a relatively small sample size, and was not directly comparable, the detection yields of TXI1 and TXI2 were generally consistent with those of these RCTs.

Although TXI demonstrated higher visibility than WLI, TXI2 lacks color enhancement and is considered less visible than TXI1. One report showed significantly lower visualization scores for TXI2 than for TXI1 [14]. Nevertheless, our results did not demonstrate a difference in the detection yields of TXI1 and TXI2. One possible explanation for this is that these visibility studies were based on still images, which may not fully reflect clinical practice. Although TXI1 may be familiar to endoscopists because its imaging resembles linked color imaging (LCI), TXI2 produces images closer to those of WLI and may therefore offer higher user‐friendliness in routine practice.

Additionally, to investigate which adenomatous lesions were more likely to be detected per patient in each observation mode, we analyzed the MAP based on morphology and tumor size. In the morphological analysis of MAP, non‐polypoid lesions and flat polyps were detected more frequently in both the TXI1 and TXI2 groups. Previous studies have indicated that non‐polypoid or flat lesions, including the laterally spreading tumor non‐granular type (LST‐NG), may be overlooked during WLI endoscopy because of their subtle appearance and low contrast with the surrounding mucosa [8, 32]. These overlooked lesions are at risk of being detected as PCCRC in the future. According to a recent large‐scale study [33], among non‐polypoid lesions, the LST‐NG has been mentioned as having the potential to be a precursor to PCCRC. Additionally, advanced neoplasia detected during surveillance endoscopy mainly consists of LST‐NG lesions, and a history of LST‐NG resection has been reported to be a risk factor for advanced neoplasia. One method for avoiding missing or detecting flat lesions, including LST‐NGs, is IEE, such as narrow‐band imaging (NBI) and LCI. In a prospective study comparing NBI and WLI, the number of flat and depressed lesions detected using NBI was significantly higher than those detected using WLI [7]. In contrast, according to a meta‐analysis comparing LCI and WLI, the number of flat lesions detected using LCI was significantly higher than those detected using WLI [10]. IEE, which can enhance lesion contrast, may excel in the depiction of non‐polypoid and flat lesions. Additionally, TXI1 and TXI2 are expected to have detection capabilities comparable with those of NBI and LCI, respectively.

The strength of our post hoc analysis was that we evaluated the diagnostic yields of both TXI1 and TXI2 using real‐world data. In many RCTs comparing IEE and WLI [6, 8, 9, 34], the necessity of maintaining trial quality often results in examiners being experts. Our study, which is a retrospective analysis in clinical practice, determined the detection yield of TXI among non‐experts. Notably, among the non‐experts, the use of TXI1 or TXI2 achieved both comparable ADR and FDR values, suggesting that even non‐experts can detect flatter lesions with high malignant potential using TXI.

Recently, an RCT conducted by Toyoshima et al. did not demonstrate the superiority of TXI1 over WLI when MAP was used as the primary outcome [35], although other studies, including meta‐analyses, have reported that TXI significantly improves the detection of colorectal adenomas [16, 30, 31, 36]. The detection yield of TXI in the RCT was sufficient, but the differences from previous reports may be explained by the improved performance of WLI resulting from the combination of the EVIS X1 system and the CF‐EZ1500DI scope, as well as the differences in primary endpoints and study populations.

As mentioned in our original cohort [18], similar limitations exist in this post‐hoc analysis. The primary limitation was a potentially insufficient sample size. As we divided the TXI into TXI1 and TXI2 groups, resulting in smaller numbers in each group, these results should be interpreted with caution. Second, a facility bias may have occurred. This study was conducted only in tertiary centers in Japan, which may not reflect the environment of community hospitals. Therefore, the results may not be generalizable to the general population undergoing colonoscopies. Finally, this was a post hoc analysis based on data from our previous multicenter observational study. Although the dataset was the same, the present analysis focused specifically on comparing the clinical performances of TXI1 and TXI2, which were not evaluated in the original report. Therefore, this analysis should be considered a supplementary investigation aimed at generating new insights relevant to real‐world clinical practice.

In conclusion, our post hoc analysis revealed that TXI1 and TXI2 demonstrated comparable detection yields for colorectal lesions. In clinical practice, endoscopists may select either mode according to their experience, preference, or institutional practice without substantial disadvantages. Accordingly, the necessity for large‐scale trials that directly compare TXI1 and TXI2 is limited. Future studies should focus on clarifying the role of TXI in comparison with other image‐enhanced endoscopy modalities, such as NBI and LCI, and evaluate its usefulness in educational settings and procedures performed by non‐experts.

## Author Contributions

**Study conception and design**: Hiroaki Ikematsu, Taku Sakamoto, and Yutaka Saito. **Data acquisition**: All authors. **Data analysis**: Kensuke Shinmura, Hiroaki Ikematsu, and Taku Sakamoto. **Data interpretation**: All authors. **Manuscript drafting**: Kensuke Shinmura. All the authors reviewed and critically revised the manuscript. All the authors approved the final version of the manuscript.

## Conflicts of Interest

Yutaka Saito is the director of DEN Open and has received lecture fees from Olympus Corporation; Kensuke Shinmura is a deputy Editor‐in‐Chief of DEN Open; and Hiroaki Ikematsu is an associate editor of DEN Open. This study was supported by the Olympus Corporation as a joint research organization of the three institutions. The authors declare no conflicts of interest.

## Funding

This study was funded by the Olympus Corporation. The funder played no role in the design, data collection, data analysis, or reporting of this study.

## Ethics Statement

The study protocol was reviewed and approved by the National Cancer Center Hospital Certified Review Board (approval number: 2020‐313). This study was conducted in accordance with the Japanese Ethical Guidelines for Medical and Health Research on Human Subjects.

## Consent

Informed consent was obtained from the participants in each institution using the opt‐out method.

## Clinical Trial Registration

N/A

## Data Availability

Individual participant data will not be shared.
